# Psychological capital and climate change adaptation: Empirical evidence from smallholder farmers in South Africa

**DOI:** 10.4102/jamba.v13i1.1061

**Published:** 2021-06-28

**Authors:** Unity Chipfupa, Aluwani Tagwi, Edilegnaw Wale

**Affiliations:** 1 Department of Agriculture and Animal Health, School of Agriculture and Life Sciences, University of South Africa, Johannesburg, South Africa; 2 Department of Agricultural Economics, Faculty of Natural and Agricultural Sciences, University of the Free State, Bloemfontein, South Africa

**Keywords:** psychological capital, smallholder farmers, climate change, adaptation, non-cognitive factors, adaptive capacity, multivariate probit

## Abstract

There are calls for better empirical models to inform climate change adaptation in smallholder agriculture. Hitherto adaptation studies have failed to comprehensively integrate non-cognitive behavioural factors (e.g. psychological capital), and there is also no common framework for measuring non-cognitive abilities of smallholder farmers. Hence, this study is the first attempt to assess how psychological capital affects climate change adaptation amongst smallholder farmers. The study estimated the multivariate probit regression model using data collected from 328 smallholder farmers in KwaZulu-Natal province, South Africa. The results show an association between some psychological capital indicators and smallholder adaptation decisions. Social networks, having multiple farming objectives, access to credit and the type of farmer (irrigators vs. non-irrigators) were also significant in determining smallholders’ adaptation decisions. In conclusion, the study recommends the need for practical ways for enhancing smallholders’ endowment with key non-cognitive abilities. There is also a need for researchers to develop a comprehensive framework for assessing non-cognitive factors critical for climate change adaptation. This will improve the use of positive psychology theories to advance the literature on climate change adaptation. Support should also be provided to communities facing higher risks of climate change adaptation. More focus should also be given to improve smallholder farmers’ ability to adapt, including access to affordable credit. The role of social networks in information sharing remains critical, and hence their promotion should be prioritised. The findings on multiple objectives in farming were unique to climate change adaptation research, and hence the indicator should be considered in future similar studies.

## Introduction

Climate change remains one of the world’s critical challenges and a key factor hindering progress in achieving the Sustainable Development Goals (IFPRI 2019). For instance, the ever-increasing frequency and magnitude of climate change-related disasters in the United States of America are making the country pay a hefty price. Projections suggest that by 2050, climate change will result in a 3% – 6% decline in the global food production and a 0.28% loss in the global gross domestic product (Calzadilla et al. 2013). In the same period, the yield of cereal crops in Africa will likely reduce by between 5% and 17%, with adverse impacts on food security and livelihoods (Knox et al. 2012). The Intergovernmental Panel on Climate Change models are projecting that climate change will result in cereal price increases of between 1% and 29% by 2050, and also a reduction in the nutritional quality of food (Mbow et al. 2019). The impact will be most felt in developing countries because of their agriculture-based economies. Hence, regions like sub-Saharan Africa cannot afford to ignore climate change and adaptation remains a necessary response. However, there are calls for better models to inform climate change adaptation efforts, especially amongst smallholder farmers (Swim et al. 2009; Wuepper, Zilberman & Sauer 2019).

Climate change adaptation in the agricultural sector has widely been studied (Burnham & Ma 2016; Islam & Nursey-Bray 2017; Khanal et al. 2018; Trinh et al. 2018; Truelove, Carrico & Thabrew 2015; Wuepper et al. 2019). However, there has been an unbalanced focus on the thematic areas known to influence human adaptive behaviour to climate change. Most studies have focused on tangible factors (e.g. demographic factors, assets, institutions and social networks), neglecting the intangible non-cognitive behavioural factors (e.g. personality traits, attitudes and motivation) (Dang et al. 2019; Grothmann et al. 2013; Truelove et al. 2015). Dang et al. (2019) posited that researchers shy away from such factors because they tend to be contextual, complex and challenging to measure. However, there are studies that have demonstrated the importance of non-cognitive factors to climate change adaptation (Swim et al. 2009; Truelove et al. 2015; Wuepper et al. 2019), economic decisions (Lybbert & Wydick 2018), irrigation farming (Chipfupa & Wale 2018b; Phakathi & Wale 2018), disaster resilience and hazard risk perception (Armaş, Cretu & Ionescu 2017; Béné et al. 2019; Mertens et al. 2018). The conclusions of these studies show that non-cognitive (psychological) factors influence the decisions of individuals or households and hence their adaptive capacity to stressors in life. Thus, the failure to comprehensively account for these factors will likely result in the design of inappropriate climate change adaptation policies and strategies (Feola et al. 2015).

Adaptation to climate change in smallholder agriculture refers to the farm household’s ability to develop practical ways for reducing the impacts of climate change events, such as drought, floods, hailstorms, heat waves and strong winds, amongst others (Grothmann & Patt 2005). It requires both the ability and willingness to adapt. While ability is a question of endowment with livelihood assets, willingness is by and large a behavioural question. Climate change adaptation is a behavioural aspect that is influenced by one’s decision-making (Feola et al. 2015; Grothmann & Patt 2005). Decision-making itself is a psychological construct because it involves a non-cognitive process of applying scientific knowledge to the selection of a course of action amongst many alternatives (Todt & Luján 2014).

This article is the first attempt to empirically examine the role of psychological capital in climate change adaptation. Psychological capital is a form of non-cognitive skill which defines an individual’s mindset that determines his or her propensity to make the right decisions and choices in life (Luthans, Luthans & Luthans 2004). It is the primary asset that can explain why individuals endowed with the same resources and working environment can perform differently, a feature common amongst smallholder farmers. It is also the ultimate asset that determines the effective and efficient use of all the other resources that an individual or household possesses (Chipfupa & Wale 2018b). The literature has distinguished four dimensions that constitute psychological capital, that is, self-confidence (internal locus of control), optimism, hope and resilience (Luthans et al. 2004; Luthans, Youssef-Morgan & Avolio 2015). According to Luthans et al. (2015), self-confident individuals have a belief in their ability to accomplish something, even in the presence of challenges. Optimism allows such individuals to take challenges as opportunities and look forward to a better future, while hope affords them the willpower to explore different routes of addressing such challenges. Resilience gives them the ability to cope with adversities. Given these constructs, the article argues and postulates that psychological capital affects not only smallholder farmers’ response to climate change but also their demand for adaptive strategies.

Developments in the field of positive psychology have demonstrated that it is possible to provide a standardised measure of the psychological capital endowment of an individual. Studies that have attempted to integrate the effect of non-cognitive factors in climate change adaptation research (Dang et al. 2014; Grothmann & Patt 2005; Mertens et al. 2018; Truelove et al. 2015; Wuepper et al. 2019) fail to provide such a comprehensive measure that captures all the facets of psychological capital. There are also no generic indicators for measuring non-cognitive abilities of smallholder farmers, with each study developing their own construct. Like many other psychosocial studies, the ones mentioned above are mostly influenced by Bandura’s self-efficacy theory (Bandura 1977) and the locus of control theory (Rotter 1966). The challenge, however, is that the two theories have a weakness because of their inability to address the other non-cognitive aspects related to hope (willpower to accomplish) and resilience (perceptions on the ability to adjust and adapt). This study proposes the application of a more comprehensive and robust theory, the ‘psychological capital theory’ (Youssef-Morgan & Luthans 2013). To the best of the authors’ knowledge, no other study has applied this theory in its entirety to climate change adaptation research. This is the main contribution of the study.

The article builds on recent attempts (Chipfupa & Wale 2018b, 2020; Phakathi & Wale 2018) to integrate psychological capital to the sustainable livelihoods framework and measure it using stated preference-based questions meant to capture the above dimensions. Using empirical survey data, it assesses how psychological capital affects climate change adaptation amongst smallholder farmers in rural KwaZulu-Natal, South Africa. Hence, this study employs a multivariate probit model (mvprobit) to assess the effect of psychological capital indicators on the adoption decisions of different climate change adaptation strategies.

## Psychological capital and adaptive behaviour: The conceptual link

Adaptation behaviour has been conceived in the climate change literature using the protection motivation theory (PMT) (Grothmann & Patt 2005; Mertens et al. 2018; Swim et al. 2009; Truelove et al. 2015). The theory states that people facing a threat will adopt behaviours that protect themselves if they deem the risk of the threat to be high (Rippetoe & Rogers 1987; Rogers 1983). If the loss as a result of the risk is deemed to be lower than the cost of adapting, they are expected to maintain the *status quo*. Otherwise, they will adapt. Some similarity exists between the PMT and the random utility theory (RUT). According to the RUT, farmers generally choose what they prefer, and that preference considers the utilities of the different options. Where they do not do so, their choice can be explained by random factors (Cascetta 2009). This study draws from the same literature, extending the work of Grothmann and Patt (2005) and Truelove et al. (2015).

Grothmann and Patt (2005) and Truelove et al. (2015) proposed models for assessing the effect of psychological factors on adaptive behaviour. Their models discuss a non-cognitive process of risk and adaptation appraisal that results in climate change risk perceptions and perceptions on adaptive capacity. However, they do not explicitly present what constitutes a non-cognitive process. This study posits that people’s risk and coping appraisals are themselves a result of an underlying psychological construct, here referred to as psychological capital. Béné et al. (2019) also proposed a similar psychosocial conceptual framework for assessing resilience capacities of households in disaster crises. However, their focus was on how psychosocial factors affect people’s ‘subjective resilience^1^’ and hence their responses to disasters. Again, this is only one dimension of psychological capital.

**FIGURE 1:**
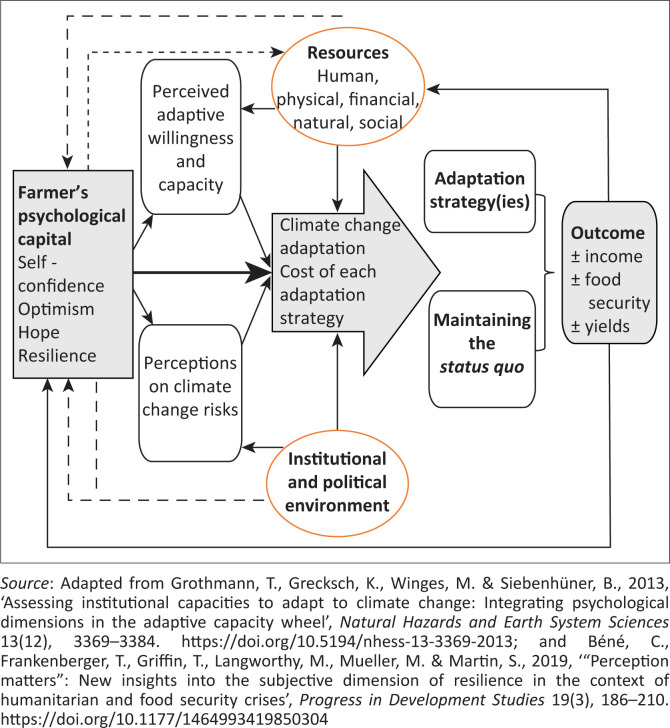
The psychological capital model of adaptive behaviour to climate change.

Figure 1 depicts a comprehensive psychological capital model of adaptive behaviour to climate change. It starts by highlighting the four constructs of psychological capital which potentially affect the perceptions of smallholder farmers about any shock they face (e.g. climate change; perceived climate change risks) and their adaptive capacity (perceived adaptive willingness and capacity) (Béné et al. 2019; Grothmann et al. 2013; Wuepper et al. 2019). The constructs affect smallholder farmers’ adaptation motivation, that is, the realisation of the climate change risk and the need for action (Grothmann et al. 2013). A farmer endowed with positive psychological capital is better placed to make a value judgement about the perceived probability and severity of a climate change threat. Likewise, if they do not perceive to have the ability to be resilient, they would not make adaptation decisions.

Once an individual has formulated an intention to adapt, their ability and extent of response (adaptation strategies) will depend on four key factors, that is, their resource endowment, cost of each adaptation strategy, perceived risk of maintaining the *status quo* and the institutional and political environment. Regarding resource endowment, including psychological capital, it is envisaged that the propensity of smallholder farmers to act will be a function of their willingness (psychological readiness to face the opportunity cost of adaptation) and their ability (asset endowment). Each adaptation strategy has a cost implication to be borne by the farmer. The challenge is that more often than not smallholder farmers find themselves constrained by resources and the operating environment that affects their ability to adapt (Veider & Matzler 2016). However, the final decision will depend on the perceived risk of not taking any action. The higher that risk, the higher their motivation to adapt and vice versa. The institutional environment defines the rules, value systems and regulations existing in society (Swaminathan & Wade 2016). The key functions of institutions in smallholder farming include information provision, capacity building and facilitating access to finance and markets. Several studies have shown that institutions influence the adaptive capacity to climate change (Berman, Quinn & Paavola 2012; Mubaya & Mafongoya 2017).

In practice, differences will be observed among farmers in terms of psychological capital endowment and their perceived adaptation efficacy (the belief in one’s ability to respond to the climate change threat). Irrespective of the perceived cost of adaptation and existing institutions, smallholders with similar resources might respond differently to a climate change threat. In other words, their willingness to adapt, which constitutes their objective orientation and motivations (Veider & Matzler 2016), will differ by the level of their psychological capital endowment. Those with a higher level of internal locus of control are inclined to organise their endowments to protect themselves from the climate change threats, while others (with an external locus of control) wait for external support (from government or other entities) to bail them out.

## Research methodology

### Data collection and sampling

**FIGURE 2:**
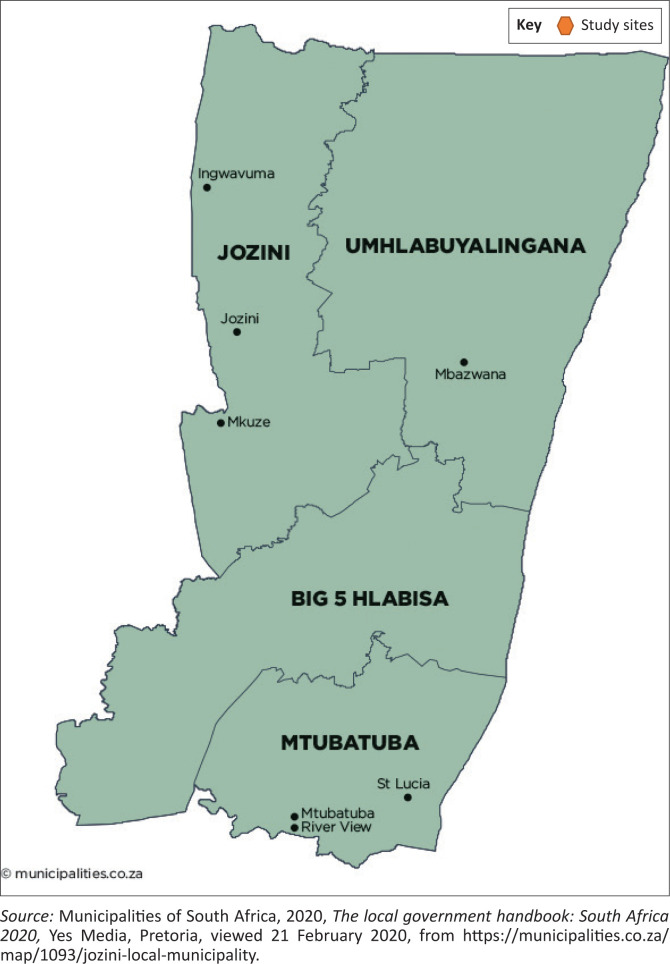
Jozini local municipality.

The survey was conducted in 2016 in Jozini, a local municipality in the northern part of KwaZulu-Natal province, South Africa (see map in Figure 2). It was part of a Water Research Commission project meant to identify the smallholder entrepreneurial development pathways, taking advantage of the irrigation schemes in KwaZulu-Natal. Jozini has a population of 198 215, 55% being between the ages of 15 and 64 years (Statistics South Africa 2016). Agriculture is a key economic sector in the municipality. However, it is being affected by climate change. The larger part of the municipality has a mean annual rainfall of 600 mm. Temperatures in the summer can be as high as 40 °C, while average evapotranspiration is 1660 mm and is highest in the winter (Jozini Local Municipality 2017). Hence, drought and high temperatures are common climate change outcomes in the municipality. This makes irrigation farming important to the livelihoods of smallholder households in this community. The spatial development framework of Jozini acknowledges the need for development interventions to consider climate change in their conception, design and implementation.

The study targeted 328 smallholder farmers in and around two irrigation schemes, Makhathini and Ndumo-B. A multistage sampling technique was employed in identifying the farmers. The first stage involved identifying the municipality for the project, which was done purposively to obtain areas with potential in both irrigation and rainfed agriculture. The two main schemes and their surrounding communities (within the 10 km radius) in the selected local municipality were then targeted for the survey. All 21 scheme farmers in Ndumo-B and 10% of those in Makhathini (109) were interviewed. Random sampling was used for scheme farmers in Makhathini using the list of smallholders provided by the scheme management. The farmers from the surrounding communities were identified through snowballing because of lack of prior information. A sampling interval of three households was used to ensure representativeness in the sample. The study questionnaire and procedures were approved by the Human and Social Sciences Research Ethics Committee of the University of KwaZulu-Natal (protocol reference number: HSS/0601/015D), and informed consent was obtained from the respondents. More details about the data collection procedures can be found in Chipfupa (2018).

### Analytical framework

#### Multivariate probit model

Recent adaptation studies have recognised the need to account for the interdependence of household adaptation decisions (Mulwa, Marenya & Kassie 2017; Ojo & Baiyegunhi 2019). This means that the adaptation strategies can be used simultaneously. Failure to account for such interdependence may result in biased estimates and wrong conclusions (Danso-Abbeam & Baiyegunhi 2017; Greene 2012). The mvprobit model estimates more than one binary probit equation simultaneously, allowing the error terms to be correlated (Greene 2012). This is not possible with the univariate probit model. Thus, the mvprobit model was estimated to determine the effect of psychological capital on different adaptation decisions of smallholder farmers.

Following Greene (2012), the study formulates the mvprobit model with three binary dependent variables (Pd, Dr and Sc) as follows:

Let $y_{im}^{*}$ in Equation 1 be the latent variable that contains both observed and unobserved preferences associated with the *m*th climate change adaptation strategy. Then,

$$y_{im}^{*}=\beta_{m}x_{im}^{'}+\varepsilon_{im},\;where\;m=Pd,\;Dr\;and\;Sc$$ [Eqn 1]

$$y_{im}=1\;if\;y_{im}^{*}>0,\;0\;otherwise,$$ [Eqn 2]

where *Pd, Dr* and *Sc* represent the strategies, that is, changed planting dates, planted drought-resistant or short-season crops and soil conservation strategies, respectively. $x_{im}^{'}$ is a vector of explanatory variables. The error terms from the three outcome variables, ε*_im_*, from Equations 1 and 2, are interdependent and are multivariate normally distributed with a mean of 0 and a variance of 1. This means that a household can make simultaneous decisions and choose more than one strategy at any given time. The variance–covariance matrix (Ω) of Equation 1 is symmetrical and has values of one on the leading diagonal and pairwise correlation (ρ) of the error terms of the three outcome variables (e.g. *ρPdDr, ρDrSc)* as off-diagonal elements (Eqn 3). This assumption allows for the joint estimation of the different adaptation decisions (Kassie et al. 2013):

$$\Omega =\left[\begin{array}{ccc} 1 & \rho PdDr & \rho PdSc\\ \rho DrPd & 1 & \rho DrSc\\ \rho ScPd & \rho ScDr & 1 \end{array}\right]$$ [Eqn 3]

The reduced form of the mvprobit estimated in this study is thus given by Equation 4:

$$y_{im}=\beta_{0}+\sum_{i=1}^{k}\beta_{im}F_{im}+\sum_{i=1}^{k}\beta_{im}H_{im}+\sum_{i=1}^{k}\beta_{im}Ps_{im}+\sum_{i=1}^{k}\beta_{im}I_{im}+\sum_{i=1}^{k}\beta_{im}L_{im}+\varepsilon_{im}$$ [Eqn 4]

where ∀*i* = 1…‥*k* regressors and *β*s are parameter estimates. *F*_*i*_, *H*_*i*_, *Ps*_*i*_, *I*_*i*_ and *L*_*i*_ represent variables for household characteristics, household asset variables, psychological capital indicators, institutional variables and other variables (such as location and type of farmer), respectively.

#### Independent variables

Table 1 presents the explanatory variables included in the empirical models. Psychological capital endowment was expected to affect smallholder farmers’ response to climate change positively. Three psychological capital indicators were included as predictor variables. These were ‘confident, optimistic and hopeful’, ‘resilient’ and ‘venturesome and future-focused’. The three are principal components (PCs) obtained through an approach informed and well documented in papers published by Chipfupa and Wale (2018b) and Phakathi and Wale (2018). The methodology involved the collection of data on 12 five-point Likert-scale questions (three for each psychological capital construct). The Likert-scale responses were subjected to a principal component analysis, which resulted in three PCs with an eigenvalue of more than 1 (see results in Appendix 1). The first PC represented farmers possessing most of the dimensions of psychological capital, that is, self-confidence, optimism and hope. The second PC represented resilient farmers with high levels of self-confidence and risk-taking tendencies. The third PC represented adventurous and future-focused smallholders, not afraid to explore new opportunities.

**TABLE 1:** Description of independent variables included in the model.

Variables	Variable description	Mean	Standard deviation	%	Expected sign
**Continuous variables**					
AGE	Age of household head (years)	48.90	11.91	-	+
AGESQ	Square of age of household head	2532.48	1144.78	-	‒
EDU_LEVEL	Schooling (years)	4.28	4.50	-	+
DEP_RATIO	The proportion of dependents over productive members	0.91	1.03	-	±
EXPERIENCE	Years in farming	13.80	10.95	-	±
SOCG	The proportion of income from social grants	0.56	0.74	-	‒
LAND_AREA	Land in hectares	1.66	3.14	-	+
ASSETS	Log of the total value of assets	3.51	0.64	-	+
MKT_DIST	Walking time to the nearest town (minutes)	15.76	13.96	-	‒
CONF_OPT_HOPE	PC-generated – Self-confident, optimistic and hopeful	0.00	1.00	-	+
RES_CONF_RISK	PC-generated – Resilient, confident and risk-taking	0.00	1.00	-	+
VENT_FUTURE	PC-generated – Venturesome and future-focused	0.00	1.00	-	+
COOP_SOCIAL	PC-generated – Membership in cooperatives and social groups	0.00	1.00	-	+
**Dummy variables**					
GENDER_FR	Gender of household head (1 = male and 0 = otherwise)	-	-	35.10	+
MULTI_OBJ	Multiple objectives (1 = multiple objectives, 0 = otherwise)	-	-	33.80	+
ACCESS_CREDIT	Access to credit (1 = access credit and 0 = otherwise)	-	-	38.10	+
EXTENSION	Access to extension services (1 = access extension, 0 = otherwise)	-	-	74.00	+
TYPE_FR	Type of farmer (1 = non-irrigator, 0 = otherwise)	-	-	84.00	+
LOCATION	Location (1 = Makhathini, 0 = otherwise)	-	-	65.85	±

Note: Dummy variable figures are percentages of category 1. Because of normalisation, all PCs have a zero mean and unit variance.

Several variables (gender, age, the square of age, dependency ratio, proportion of income from social grant and multiple objectives) were included to control for heterogeneity in the household characteristics of the smallholder farmers. Most of the respondents were women (64.9%). The average age of the farmer was 49 years. Age was expected to have a positive relationship with the adoption of climate change adaptation strategies. However, after some cut-off point, age could also be associated with economic inactivity and a lack of willingness to change or adapt to new realities (Danso-Abbeam & Baiyegunhi 2017). Hence, the square of age was included in the model. The average year of schooling was 4.28 years, which shows low levels of education amongst smallholder farmers in the study areas. Given the importance of education in human capital development, adoption of adaptation strategies was expected to be positively influenced by more years of schooling.

The dependency ratio directly measures the pressure on the productive members of the household and indirectly provides a proxy of the labour availability. It affects the ability of the household to respond to any shocks, including climate change. The expected influence of this variable on adoption is indeterminate. This is because, on the one hand, more dependents could drive households to adopt adaptation strategies that will lessen the economic pressure on the productive members. On the other hand, fewer productive members could negatively affect the adoption of more labour-intensive strategies. A dummy variable for multiple objectives in farming was included in the model. Farmers engage in farming for different reasons, that is, subsistence only (31.8%), income generation only (34.3%) and multi-objectives (subsistence, income generation and employment) (33.8%). Smallholder farmers with multiple objectives in farming were expected to have a higher adaptive capacity.

One of the lessons from behavioural economics is that the frequency of past behaviour influences current behaviour (Dawnay & Shah 2005). The frequency of free handouts (inputs, services and social grant) by government departments and other development partners can entrench dependency and entitlement behaviour amongst recipients (Aliber 2019; Mertens et al. 2018; Sinyolo, Mudhara & Wale 2017). That behaviour, in turn, could reduce farmers’ willingness and incentives to adapt to climate change. Thus, the proportion of the household income coming from social grants was included to assess the impact of social protection policies on farmers’ response to climate change. The household asset indicators (log of the estimated total asset value and land available for farming) were expected to be positively associated with the adoption of climate change adaptation strategies. The assets (physical and livestock) in a household are indicators of their wealth status and resource availability. Similarly, land is an essential asset in farming and studies have shown that its shortage could constrain smallholder farmer response to climate change (Kassie et al. 2013; Mulwa et al. 2017).

Three variables (access to credit, access to extension and distance to market) were included as proxies for institutional support services available to the farmers. All, except distance to market, were expected to have a positive influence on climate change adaptation decisions. Access to credit was low (only 38.1% received credit), and most of it was obtained from informal micro-lenders (*mashonisas and stokvel*) at high interest rates, between 30% and 60% per month (Mashigo 2012). *Mashonisas*, also informally known as loan sharks, are individuals who lend money for profit. *Stokvels* are informal saving and lending clubs. Although formal credit sources were available (commercial banks and microfinance institutions), their stringent credit requirements made them inaccessible to smallholder farmers. About 74% of the farmers reported that they had access to agricultural extension. However, further discussions showed an asymmetry in access to extension services between those in irrigation and rainfed farming. Smallholder farmers in Makhathini irrigation scheme complained of inadequate and ineffective extension services. The other variable taken as a proxy for social capital was membership in social groups. Most of the farmers were members of different social groups that exist in the community (cooperatives – 67%, commodity groups – 8% and other community groups – 52%). These groups act as platforms for sharing information, experiences and learning, and also for receiving support services (especially the cooperatives).

Other variables included as predictor variables are dummies for the type of farmer and the location. About 86% of the farmers were engaged in different forms of irrigation farming, that is, scheme irrigation, independent irrigation (surrounding the schemes) and homestead gardening (irrigating small gardens near their homes and back yards). Only a few (14%) were still in rainfed or dryland farming, and these face enormous water scarcity challenges because of frequent drought and inconsistent rainfall.

## Results and discussion

### Occurrence of climate change events and smallholder farmers’ response

**FIGURE 3:**
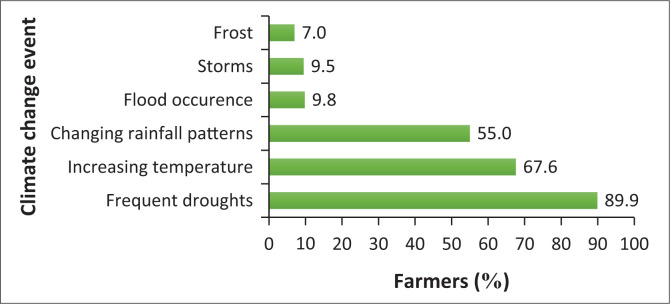
Common climate change events experienced by smallholder farmers.

The common climate change phenomena reported by smallholders in Jozini are frequent droughts (89.9%), increasing temperature (67.6%) and changing rainfall patterns (55%) (Figure 3). All three effects are inter-related and are not unique to South Africa but are experienced across the Southern African Development Community (SADC) region (Ndlovu, Prinsloo & Le Roux 2020). KwaZulu-Natal province has been experiencing drought since 2013, albeit at different scales. The worst years which received below normal rainfall were 2014/2015 and 2015/2016 (KZN DARD 2019). However, the negative effect of these events on smallholder farmers has been immense. This is mainly because of their poverty, vulnerability or susceptibility to any shock and dependence on rainfed farming to supplement their livelihoods. A few farmers also mentioned flood, storms and frost.

**FIGURE 4:**
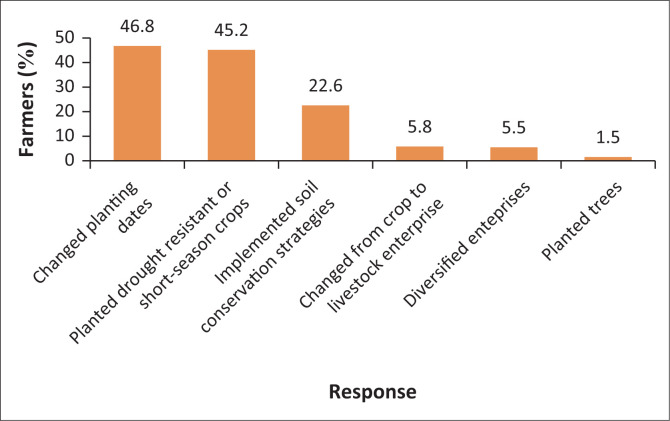
Smallholder farmers’ response to climate change.

Figure 4 shows that smallholder farmers’ response to the above climate change effects is in the form of mainly three adaptation strategies: changing planting dates, planting drought-resistant or short-season crops and implementing soil conservation strategies. These can be implemented in the short to medium term. The three strategies are a direct response to the water shortage problem. Hence, it can be concluded that the impact of climate change (mainly drought) on water availability, which is the source of risk to farmers’ livelihoods, is an incentive for them to take action. The results are similar to those found by Tessema, Joerin and Patt (2019). Several studies have shown that early or late planting increases rainfall availability to the crop and hence its chances of survival (Acharjee et al. 2019; Nouri et al. 2017). Nouri et al. (2017) found that the use of drought-resistant varieties increased maize productivity under different conditions of climate change. The adoption of short-season varieties will also reduce the water requirements of the crop and its exposure to other hazards (floods, storms, pest and drought), thereby increasing performance. As climate change also increases soil erosion, soil conservation techniques (such as conservation tillage), have also been found to reduce climate change impact on the soil (Garbrecht et al. 2015). Rainfed farmers also indicated irrigation farming as a long-term adaptation option. However, this depended on the availability of irrigable land either near a water source or river or in the irrigation schemes, which is not guaranteed.

### Model specification tests

Table 2 shows pairwise correlations from the mvprobit estimation. The estimation had three dependent variables (the three common strategies in Figure 4). The results showed that the correlations of all the error terms were significant and positive. The likelihood ratio (LR) test that assesses the overall relationship between the three adaptation strategies also showed a significant result, χ = 223.02; *p* > 0.000, (rejecting the null hypothesis of no correlation). The Wald test for joint significance of the adaptation strategies was also significant (χ = 100.35; *p* > 0.001). This shows that the adaptation decisions for the three strategies are interdependent and hence should be estimated jointly. The joint probability of success (that the farmers will adopt all of the three strategies) was 20%, while that of failure (none of the farmers will adopt all of the three strategies) was 43%. The positive coefficients of the error terms show a complementary relationship between each pair of the adaptation strategies. Overall, the results show that the use of the mvprobit for estimating the model was appropriate. Checks for multicollinearity were performed using the variance inflation factor (VIF). The results showed no presence of multicollinearity with a VIF of 6.52, which is below the threshold value of 10.

**TABLE 2:** Pairwise correlation matrix from the mvprobit estimation.

Variable	*P_j_*	Dr		Sc		Chi^2^(3)	*p*
		ρ	Standard error	ρ	Standard error		
Pd	-	0.914†	0.025	0.752†	0.061	-	-
Dr	-	-	-	0.776†	0.062	-	-
LR test	-	-	-	-	-	478.96	0.000
Success	0.201	-	-	-	-	-	-
Failure	0.430	-	-	-	-	-	-

*P_j_*, joint probability; Sc, soil conservation strategies; Pd, changed planting dates; Dr, planted drought resistant or short-season crops; LR, likelihood ratio.

† , Significantly different at 1%.

### Model results

Several factors appear to determine climate change adaptation decisions of smallholder farmers (see Table 3). Two of the three psychological capital factors, that is, CONF_OPT_HOPE and VENT_FUTURE, have a positive and significant relationship with smallholder farmers’ climate change adaptation decisions. The results suggest a considerable association between psychological capital factors and climate change adaptation behaviour. This is consistent with findings from other studies (Abay, Blalock & Berhane 2017; Truelove et al. 2015; Wuepper et al. 2019) and confirms the importance of the non-cognitive factors in climate change adaptation. However, the fact that psychological capital variables are only significant in four out of the nine possible outcomes means that the effect of the different constructs cannot be assumed but should always be assessed against the decisions that farmers make. It also means that the psychological capital concept is relevant within the context which the smallholder farmers are making the adaptation decisions. Certainly, the factor ‘RESILIENT’ appears to have minimum influence on the adaptation decisions of the sampled farmers. This is because resilience requires both mental or emotional strength and ability (Béné et al. 2019). As noted earlier, ability is a function of resources that an individual possesses. Hence, subjective resilience alone without the personal assets for translating the non-cognitive skill into tangible coping mechanisms has little or no effect on the climate change adaptation decisions of farmers.

**TABLE 3:** Multivariate probit regression model estimation results.

Variables	Changed planting dates		Drought-resistant or short-season crops		Soil conservation strategies	
	Coefficient	Standard error	Coefficient	Standard error	Coefficient	Standard error
GENDER_FR	-0.311*	0.179	-0.193	0.177	0.140	0.184
AGE_FR	0.004	0.045	-0.003	0.047	0.016	0.048
AGESQ	0.000	0.000	0.000	0.000	0.000	0.000
EDU_LEVEL	0.012	0.022	-0.019	0.022	-0.001	0.023
DEP_RATIO	-0.048	0.077	-0.032	0.075	-0.057	0.101
SOCG	0.028	0.050	0.016	0.040	0.013	0.010
CONF_OPT_HOPE	0.165**	0.080	0.182**	0.081	0.087	0.088
RESILIENT	-0.009	0.079	-0.021	0.078	0.104	0.082
VENT_FUTURE	0.064	0.081	0.157*	0.084	0.157*	0.088
EXTENSION	0.121	0.189	0.163	0.189	-0.299	0.187
ACCESS_CREDIT	0.314**	0.162	0.266*	0.163	0.269*	0.166
COOP_SOCIAL	0.178**	0.083	0.171**	0.082	0.200**	0.090
MKT_TIME	-0.006***	0.006	-0.003	0.006	0.001	0.007
LAND	0.013	0.029	0.017	0.030	0.033	0.032
ASSETS	-0.148	0.125	-0.019	0.120	-0.114	0.118
MULTI_OBJ	0.369**	0.169	0.668***	0.168	0.360*	0.169
TYPE_FR	-0.737***	0.259	-0.654***	0.254	-0.714**	0.308
LOCATION	0.238	0.179	0.789***	0.179	0.173	0.191
_cons	-0.197	1.214	-0.655	1.276	-1.149	1.315

Note: Wald Chi^2^ = 100.35; Prob > Chi^2^ = 0.000; Log likelihood = -392.26, Observations = 301.

* , significant at 10%;

** , significant at 5%;

*** , significant at 1%.

The variable CONF_OPT_HOPE had a relationship with the decisions to adopt drought-resistant or short-season crops and that of changing crop planting dates. This shows that smallholder farmers’ self-confidence increases their propensity to take immediate measures to address the impact of drought or inconsistent rainfall patterns. Being optimistic and hopeful makes them perceive climate change as a challenge and not a problem. It also makes them to see themselves as part of the solution to address the challenges caused by climate change. Mertens et al. (2018) also found that households exposed to landslide disasters in Uganda were likely not to take preventative measures because of lack of self-confidence. The variable VENT_FUTURE was associated with the decisions to adopt drought-resistant or short-season varieties and implement soil conservation technologies. Venturesome and future-oriented smallholders are more likely to decide to plant drought-resistant crops and adopt soil conservation strategies as a way of adapting to climate change. As noted earlier, the indicator VENT_FUTURE represents adventurous and future-focused smallholders ready to take advantage of any available opportunities. It is derived from the psychological capital construct ‘hope’, further demonstrating the importance of this non-cognitive factor to farmers’ adaptive capacity and behaviour. This result supports the study’s earlier argument that a comprehensive approach is needed that integrates all facets of smallholders’ non-cognitive abilities. For one to have a long-term focus and be willing to try new ideas even with limited knowledge of the potential outcomes, they need the willpower to accomplish or achieve in life (Chipfupa & Wale 2018b).

The difference between the findings of this research and those from other similar studies is in the form of the non-cognitive factors found to be important to adaptation. For example, Abay et al. (2017) showed the importance of the locus of control factors, while the significant non-cognitive factors in Wuepper et al. (2019) were formulated from information about the farmers’ self-confidence, locus of control and their time preferences (regarding payoffs). These differences emanate from the way data were collected on the actual non-cognitive indicators in each study. This is testimony to the absence of a generic framework to designing and measuring smallholders’ non-cognitive abilities or skills which affects advancement in this literature. This aspect is important to note because it highlights the need to define and map the non-cognitive abilities critical to decision-making in smallholder farming, and the respective generic questions one should ask. Such questions should address all the key dimensions of non-cognitive skills, that is, self-confidence (internal locus of control), optimism, hope and subjective resilience.

The results in Table 3 also show four other factors from the mvprobit model with a significant relationship across all the three adaptation decisions, that is, membership to social groups (COOP_SOCIAL), type of farmer (TYPE_FR), multiple objectives in farming (MULTI_OBJ) and access to credit (ACCESS_CREDIT). The relationship was positive for variables membership to social groups, multiple objectives in farming and access to credit and negative for the type of farmer. Farmers who are part of social networks were more likely to adopt different climate change adaptation strategies. Social networks serve as platforms for sharing information on overcoming the effects of climate change (Below et al. 2012). Thus, they give the farmer more options for adaptation. They also influence smallholder decisions and behaviour through the demonstration effect (Chipfupa & Wale 2018a). These findings are consistent with previous studies that have shown the importance of social networks in climate change adaptation (Mulwa et al. 2017; Ojo & Baiyegunhi 2019; Roco et al. 2014).

Regarding the type of farmers, unlike our expectations, non-irrigators (rainfed farmers) were less likely to adopt the different adaptation strategies. Notwithstanding that such farmers bear the most impact of climate change. Further descriptive analysis corroborates with this result. More smallholder farmers in irrigation (40%) compared to rainfed farming (29%) have access to credit and hence resources for use in adapting to climate change. Those in irrigation receive significantly more income (R13,716) from crop sales compared to rainfed farmers (R5,796), which boosts their ability to invest in adaptation strategies. In general, smallholder farmers in irrigation schemes have more privileges and resources than rainfed farmers. Thus, even though rainfed farmers are more impacted by climate change-related shocks (such as drought), they are less inclined to adopt adaptation strategies. This suggests that adaptation at the farm level is by and large a question of ability in terms of resource endowment. Moreover, rainfed farmers’ income from farming only constitutes 22% of their total household income compared to 30% for those in irrigation. Hence, they have less incentive to take adaptation measures to stabilise their farm income. Chipfupa and Wale (2020) have shown that smallholders are motivated to invest their time and resources on an activity that significantly contributes to their livelihoods. In summary, it is not much about the impact of climate change but the ability and willingness of farmers to make certain adaptation strategies that matter most. ‘This is one of the novel findings of this study’.

The significance of the variable MULTI_OBJ shows that smallholders with multi-objectives in farming (food self-sufficiency, income generation and employment) – who are more likely to diversify than to specialise – are more likely to adopt and implement climate change adaptation strategies compared to their counterparts. In other words, their objective orientation means that they stand to lose much from the impact of climate change compared to their colleagues. Thus, their risk aversion behaviour is inducing them to adopt different climate adaptation strategies. To the best of the authors’ knowledge, none of the other similar studies (Abay et al. 2017; Truelove et al. 2015; Wuepper et al. 2019) included this variable as a factor to explain the adaptation behaviour of smallholder farmers.

Access to credit had a significant relationship with all of the climate change adaptation decisions. This is consistent with findings from other studies (Mulwa et al. 2017; Ojo & Baiyegunhi 2019). Smallholders with access to credit are more capable of investing in one or more strategies for reducing the effects of climate change. Adaptation is a costly exercise because financial resources are required to acquire the inputs, hire more labour or invest in new infrastructure (Wilk, Andersson & Warburton 2013). However, at the current interest rate of 30% – 60% per month, consumption and informal credit is expensive and, in the long run will likely make the farmers more vulnerable (Mashigo 2012). The results also show three other variables, that is, gender, location and distance to the nearest market, which were associated with only one of the adaptation decisions.

## Conclusion and policy implications

Adaptation is critical in mitigating the effects of climate change on smallholder farming. The study used data from farmers in and around two irrigation schemes in South Africa to assess to what extent psychological capital affects their climate change adaptive capacity and behaviour. The findings support arguments of a considerable association between psychological capital and smallholder farmers’ adaptation decisions. However, other factors such as access to resources could influence how some psychological factors such as subjective resilience relate to farmers’ climate change adaptation decisions. The comprehensiveness of the psychological capital theory applied in this study made it possible to identify non-cognitive factors that are associated with smallholder farmers’ adaptation decisions. Certainly, in addition to already known factors such as self-confidence and locus of control, having hope or aspirations for oneself in farming is also important in decisions meant to address the impact of climate change on water availability. Therefore, there is a need for practical ways of changing smallholder farmers’ mindsets and enhancing their endowment with such non-cognitive abilities. At the farmer’s level, this can be done through integrating the ideals of psychological capital in available platforms such as farmer field days, farmer training workshops and mentorship programmes. The learning and sharing of experiences with colleagues and experts will bolster smallholder farmers’ willpower and belief in themselves and enhance their willingness and ability to choose contextually relevant adaptation strategies.

The exploration of non-cognitive factors in agricultural studies shows the value of interdisciplinary research in offering solutions for enhancing the livelihood of smallholder farmers. However, the non-alignment of approaches for measuring non-cognitive factors in such studies makes it difficult even to compare findings. Although there is still room for improvement on the measurement of psychological capital, it offers a basis for exploring how research can adequately account for a wide range of non-cognitive factors in future similar studies. Moving forward, researchers should develop a comprehensive framework within which information on farmers’ non-cognitive abilities can be assessed.

Studies on climate change adaptation should consider including the farmer’s objectives in farming as one of the explanatory factors. Inclusion of this variable in this study has demonstrated the need for climate change programmes to identify and provide support to communities facing higher climate change risks. The differences in adaptation between rainfed farmers and irrigators show the need to focus more effort on enhancing smallholder farmers’ ability to adapt. In this regard, enacted climate change policies should balance the need to cope with impacts in the short term while building the smallholder farmers’ capacity to respond in the long term. Adaptation policies and strategies should also recognise the value of social networks in climate change responses. Promotion of social learning platforms will enhance smallholder farmers’ adaptive capacity. There is also a need to revisit smallholder financing policies and mechanisms (both state and non-state). Smallholder farmers should be protected from the profiteering behaviour of informal micro-lenders through the promotion of mechanisms (e.g. input vouchers, subsidies and value chain financing) that do not increase their vulnerability in the long run.

## Footnotes

1 Own perceptions about one’s ability to cope with shocks.

## Appendix 1

**TABLE 1-A1:** Principal component analysis results of psychological capital measures.

Psychological capital measures	PC1	PC2	PC3
**Self-confidence**			
I am confident in farming as a way of life	0.259	0.500	-0.429
I am confident in myself as a farmer	0.871	0.167	0.007
I have the power to affect the outcome of my farming	0.618	0.459	-0.015
**Optimism**			
I am optimistic about the future of agriculture in my area	0.813	0.232	0.004
I do not give up easily	0.846	0.180	0.046
I am willing to take more risks	0.461	0.619	0.053
**Hope**			
I have hope that the quality of work will get better	0.845	0.142	0.038
I am willing to forgo a profit opportunity in the short-run in order to benefit from potential profits in the long-run	0.427	-0.085	0.590
I am willing to try new ideas even without full knowledge about the possible outcomes	-0.084	0.157	0.778
**Resilience**			
I am able to cope with shocks such as drought and other natural disasters	0.486	0.133	-0.068
I would not be farming if there was a better alternative source of income	0.004	0.809	0.095
Government is responsible for the wellbeing of rural households	-0.568	0.161	-0.282
Variation (**%**)	36.500	14.230	10.320
Cumulative variation (**%**)	36.500	49.720	60.040

Note: KMO value = 0.88; Bartlett’s test of sphericity significant at 1%; only factors with loadings > 0.5 included in the explanation of the results.
